# Extra-long femoral heads as a surrogate marker for revision risk in primary total hip arthroplasty

**DOI:** 10.1007/s00402-026-06273-9

**Published:** 2026-03-30

**Authors:** Gautier Beckers, Dominic Simon, Alexander Grimberg, Yinan Wu, Arnd Steinbrück, Boris Michael Holzapfel

**Affiliations:** 1 Department of Orthopaedics and Trauma Surgery, Musculoskeletal University Center Munich (MUM), LMU Klinikum, Munich, Germany; 2 German Arthroplasty Registry (EPRD Deutsche Endoprothesenregister GmbH), Berlin, Germany

**Keywords:** Head length, Neck length, Total hip arthroplasty, XL, High volume, Revision

## Abstract

**Aims:**

The effect of femoral head length on implant survival in total hip arthroplasty (THA) has been little studied so far. Longer heads may increase taper corrosion and reflect intraoperative complexity. This study evaluated factors associated with the use of extra-long heads (≥ XL) and their impact on implant survival.

**Methods:**

We analyzed 562,001 primary THA from the German Arthroplasty Registry. Subgroup analyses were performed by hospital annual primary THA volume (≤ 250, 251–500, ≥ 501), surgical indication (primary osteoarthritis [OA] vs. femoral neck fracture [FNF]), and fixation method (cemented vs. cementless). Logistic regression identified factors associated with ≥ XL head use, and implant survival was compared between head lengths using Kaplan–Meier analysis in both subgroups and the overall cohort.

**Results:**

The use of ≥ XL femoral heads decreased with increasing hospital volume (5.4% low, 4.5% medium, 3.0% high; *p* < 0.001). Rates were higher in FNF than OA across all volumes (8.1% vs. 4.7% in low-volume hospitals; 5.0% vs. 2.7% in high-volume hospitals). Cemented fixation was independently associated with higher odds of ≥ XL head use (OR 1.14, 95% CI 1.09–1.18, *p* < 0.001), with additional predictors including male sex (OR 2.13, 95% CI 2.06–2.19), BMI ≥ 40 (OR 1.94, 95% CI 1.77–2.12), higher Elixhauser comorbidity score (OR 1.09, 95% CI 1.04–1.15), and surgery for FNF (OR 1.92, 95% CI 1.83–2.02), while treatment at high-volume hospitals was associated with lower odds (OR 0.56, 95% CI 0.54–0.58). Kaplan–Meier analysis revealed higher cumulative revision rates with ≥ XL heads (7.2% vs. 4.5% at 9 years), consistent across all subgroups.

**Conclusion:**

The use of femoral heads ≥ XL was independently associated with lower hospital THA volume, femoral neck fracture, cemented fixation, male sex, higher BMI, and greater comorbidity burden. Their implantation was also linked to higher revision rates, suggesting that ≥ XL heads may serve as a surrogate marker for increased revision risk after primary THA.

## Introduction

Total hip arthroplasty (THA) is being performed with increasing frequency and has undergone continuous advancements in recent years [1]. Except in cases of pathological anatomy, the goal is to reproduce the patient’s anatomy, leg length, femoral offset, while providing stability, and increased range of motion [2, 3].

When the definitive implant is placed, fine-tuning of offset, muscle tension, and joint stability can be achieved by selecting different head lengths.

While the role of head diameter and the advantages of larger femoral heads, such as enhanced stability and greater range of motion, have been extensively studied [2, 4, 5], the literature on the impact of head length is scarce, particularly regarding its effect on implant survival.

Only few studies have examined the association between femoral head length and taper corrosion in THA; however, available evidence suggests that longer cobalt–chromium heads may increase fretting and corrosion at the head–neck taper junction, a phenomenon often referred to as ‘taperosis’ or ‘trunnionosis,’ [6, 7] whereas this relationship has not been demonstrated for ceramic heads [8]. Such corrosion can lead to adverse local tissue reactions which can result in adverse local tissue reactions [9] and, ultimately, necessitate revision.

Although intraoperative adjustments in head length are commonly employed to optimize biomechanical parameters, the use of extra-long heads is generally avoided or reserved as a last resort because of their potential mechanical disadvantages. Thus beyond their reported association with increased taper corrosion, extra-long femoral heads may also serve as a surrogate marker for challenging intraoperative conditions, such as difficulties in achieving optimal soft tissue tension or in restoring leg length [10]. As such, they could be indirectly associated with postoperative complications, especially tension-related such as dislocation and periprosthetic femoral fractures [10].

In light of these considerations, this study aimed to determine whether established risk factors for postoperative complications, such as low surgical volume [11–13], and surgical indication such as femoral neck fracture (FNF), which is often performed after hours [14] with less preparation time and without specialized staff [15] compared to primary osteoarthritis (OA), and implant fixation method, are associated with increased use of extra-long femoral heads (≥ XL).

We also aimed to assess whether femoral heads ≥ XL could serve as a surrogate marker for intraoperative complexity and whether their use was associated with higher revision rates.

We hypothesized that: 1- Lower-volume centers, cemented implants and FNF would demonstrate greater use of extra-long femoral heads compared to high-volume centers, cementless implants and primary OA, respectively. Finally, we hypothesized that the use of extra-long femoral heads would be associated with lower implant-survival.

## Material and method

### Data collection

Data were obtained from the German Arthroplasty Registry (Endoprothesenregister Deutschland [EPRD]), The EPRD is one of the largest arthroplasty registries worldwide. Registry coverage increased progressively after its establishment, rising from 4.7% of all hip and knee arthroplasties in Germany in 2013 to 35.7% in 2015 and 64.1% in 2017, and reaching approximately 78% in 2023. Data are currently contributed by 765 hospitals across the country [16]. The EPRD collects data from three sources: participating hospitals (surgical and patient data), prosthesis manufacturers (implant details), and health insurance funds (revision surgeries, survival, and complications) [16].

### Endpoints

The primary endpoint was the frequency of ≥ XL femoral head use according to hospital volume, surgical indication, and fixation method.

The secondary endpoint was implant survival, comparing ≥ XL heads with XS–S–M–L heads.

### Study population

The study included patients who underwent primary THA between 01.11.2012, and 30.09.2023. Patients with missing perioperative data, revision hip arthroplasty, or hemiarthroplasty were excluded from the analysis.

A total of 562,001 primary THA cases met the inclusion criteria and were included in the study. Patient demographics are summarized in Table 1.

**Table 1:** Patient demographics

Variable	Total: *N* = 562,001^1^	≥ XL: *N* = 26,186^1^	XS-S-M–L: *N* = 535,815^1^	*p*-value^2^
*Sex*				< 0.001
Female	356,108 (63%)	12,147 (46%)	343,961 (64%)	
Male	205,893 (37%)	14,039 (54%)	191,854 (36%)	
*Age*	70 (62, 78)	69 (61, 77)	70 (62, 78)	< 0.001
*Body mass Index*	27.4 (24.4, 31.1)	28.0 (24.9, 32.2)	27.4 (24.4, 31.1)	< 0.001
Underweight (< 18.5)	4,504 (0.8%)	185 (0.7%)	4,319 (0.8%)	
Normal (18.5–24.99)	123,458 (22%)	4,845 (19%)	118,613 (22%)	
Pre-obese (25–29.99)	162,551 (29%)	7,307 (28%)	155,244 (29%)	
Obese 1 (30.0–34.99]	88,979 (16%)	4,448 (17%)	84,531 (16%)	
Obese 2 [35.0–39.99]	31,690 (5.6%)	1,877 (7.2%)	29,813 (5.6%)	
Obese 3 [ ≥ 40)	12,814 (2.3%)	927 (3.5%)	11,887 (2.2%)	
*Weighted Elixhauser score*	0.0 (0.0, 3.0)	0.0 (0.0, 5.0)	0.0 (0.0, 3.0)	< 0.001
( < 0)	82,164 (15%)	3,987 (15%)	78,177 (15%)	
(0–3)	344,828 (61%)	14,979 (57%)	329,849 (62%)	
(≥ 4)	135,009 (24%)	7,220 (28%)	127,789 (24%)	
*Mortality*	52,265 (9.3%)	3,126 (12%)	49,139 (9.2%)	< 0.001

^1^*n* (%); Median (Q1, Q3)

^2^Pearson’s Chi-squared test; Wilcoxon rank sum test

The study cohort was divided into two groups based on femoral head length: ≥XL heads and XS–S–M–L heads.

To assess how surgical volume influences the use of ≥ XL femoral heads, we used the number of THA procedures performed annually at each hospital as reported in the hospitals’ 2022 quality reports, together with the corresponding primary hip arthroplasty quality indicators from IQTIG (Institute for Quality Assurance and Transparency in Healthcare) listed therein. While no universally accepted definition of high- and low-volume centers exists [13, 17], we stratified hospitals into three categories based on annual primary elective THA volume: ≤250, 251–500, and ≥ 501 THA procedures per year, consistent with prior registry-based studies [18]. Comparison of patient demographics by annual hospital THA volume are summarized in Table 2.

**Table 2:** Comparison of patient demographics by annual hospital THA volume (≤ 250, 251–500, ≥ 501)

Variable	≤ 250			251–500			≥ 501		
	≥ XL *N* = 15,516^1^	XS-S-M–L *N* = 272,331^1^	*p*-value^2^	≥ XL *N* = 5,448^1^	XS-S-M–L *N* = 115,345^1^	*p*-value^2^	≥ XL *N* = 4,139^1^	XS-S-M–L *N* = 134,026^1^	*p*-value^2^
*Sex*			< 0.001			< 0.001			< 0.001
Female	7,363 (47%)	175,165 (64%)		2,548 (47%)	74,124 (64%)		1,751 (42%)	85,671 (64%)	
Male	8,153 (53%)	97,166 (36%)		2,900 (53%)	41,221 (36%)		2,388 (58%)	48,355 (36%)	
*Age*	70 (61, 78)	72 (63, 78)	< 0.001	68 (60, 76)	70 (61, 77)	< 0.001	67 (59, 75)	69 (60, 76)	< 0.001
*BMI*	27.9 (24.8, 32.1)	27.4 (24.4, 31.1)	< 0.001	28.4 (25.3, 32.4)	27.5 (24.5, 31.2)	< 0.001	28.0 (25.0, 32.1)	27.3 (24.2, 30.9)	< 0.001
*Weighted Elixhauser score*	0.0 (0.0, 5.0)	0.0 (0.0, 5.0)	< 0.001	0.0 (0.0, 3.0)	0.0 (0.0, 3.0)	0.260	0.0 (0.0, 1.0)	0.0 (0.0, 1.0)	0.457
*Mortality*	2,203 (14%)	30,182 (11%)	< 0.001	452 (8.3%)	9,073 (7.9%)	0.249	304 (7.3%)	8,091 (6.0%)	0.001

*BMI* Body mass index

*Hospital volume data were unavailable for 1,083 patients in the ≥ XL group and 14,113 patients in the XS–S–M–L group; these cases were excluded from this analysis.

^1^*n* (%); Median (Q1, Q3)

^2^Pearson’s Chi-squared test; Wilcoxon rank sum test

To minimize confounding from anatomical abnormalities that may necessitate the use of extra-long femoral heads, we isolated a cohort of patients who underwent THA for primary OA. This group was identified using ICD-10 codes M16.0 and M16.1, while excluding codes M16.2–M16.9 to avoid cases involving dysplastic, post-traumatic, or other secondary forms of coxarthrosis, where altered anatomy or increased surgical complexity may independently justify the use of XL components.

To contrast this with a non-elective clinical context in which preoperative planning is more limited, we performed a parallel subgroup analysis in patients undergoing THA for FNF (ICD-10 codes S72.00 to S72.08) (Table 3).

**Table 3:** Patient demographics of the subgroups, 1- femoral neck fracture and primary osteoarthritis

Variable	Femoral neck fracture			Primary osteoarthritis		
	≥ XL *N* = 2,460^1^	XS-S-M–L *N* = 28,943^1^	*p*-value^2^	≥ XL *N* = 17,548^1^	XS-S-M–L *N* = 399,907^1^	*p*-value^2^
*Sex*			< 0.001			< 0.001
Male Female	1,151 (47%) 1,309 (53%)	8,441 (29%) 20,502 (71%)		9,689 (55%) 7,859 (45%)	145,232 (36%) 254,675 (64%)	
*Age*	75 (67, 81)	76 (68, 82)	< 0.001	70 (62, 77)	71 (63, 78)	< 0.001
*Body Mass Index*	25.1 (22.9, 27.9)	24.6 (22.2, 27.4)	< 0.001	28.7 (25.5, 32.7)	27.7 (24.7, 31.3)	< 0.001
*Weighted Elixhauser score*	5 (0, 10)	3 (0, 8)	< 0.001	0.0 (0.0, 3.0)	0.0 (0.0, 3.0)	0.005
*Mortality*	767 (31%)	8,159 (28%)	0.002	1,574 (9.0%)	31,058 (7.8%)	< 0.001

^1^*n* (%); Median (Q1, Q3)

^2^Pearson’s Chi-squared test; Wilcoxon rank sum test

To assess the influence of implant fixation technique, we compared the use of ≥ XL heads between cemented and cementless implants, as well as across all subgroups; Because cemented stems may require preparation with a larger rasp to create an adequate cement mantle, whereas uncemented stems typically correspond closely to the final rasp size [19], we hypothesized that cemented fixation may be associated with increased use of extra-long heads. Patient demographics are summarized in Table 4.

**Table 4:** Comparison of patient demographics by implant fixation

Variable	Cemented			Cementless		
	≥ XL *N* = 4,824^*1*^	XS-S-M–L *N* = 110,588^*1*^	*p*-value^2^	≥ XL *N* = 20,114^*1*^	XS-S-M–L *N* = 413,733^*1*^	*p*-value^2^
*Sex*			< 0.001			< 0.001
Female	2,700 (56%)	84,656 (77%)		8,604 (43%)	250,415 (61%)	
Male	2,124 (44%)	25,932 (23%)		11,510 (57%)	163,318 (39%)	
*Age*	79 (74, 82)	79 (75, 83)	< 0.001	66 (58, 73)	67 (60, 74)	< 0.001
*Body mass Index*	26.6 (23.9, 30.4)	26.1 (23.5, 29.4)	< 0.001	28.4 (25.3, 32.7)	27.7 (24.7, 31.4)	< 0.001
*Weighted Elixhauser score*	3.0 (0.0, 7.0)	0.0 (0.0, 5.0)	< 0.001	0.0 (0.0, 3.0)	0.0 (0.0, 2.0)	< 0.001
*Mortality*	1,098 (23%)	19,131 (17%)	< 0.001	1,658 (8.2%)	27,226 (6.6%)	< 0.001

^1^*n* (%); Median (Q1, Q3)

^2^Pearson’s Chi-squared test; Wilcoxon rank sum test

Mortality was defined as all-cause death occurring at any time during the follow-up period, as recorded in the EPRD.

### Ethics

The EPRD is approved by the Review Board of the University of Kiel (D 473/11), and this study was conducted in accordance with the Declaration of Helsinki.

### Statistical analysis

Continuous variables are presented as median and interquartile range (Q1, Q3), while categorical variables are reported as absolute frequencies and percentages. Group comparisons between femoral head length categories (≥ XL vs. XS–S–M–L) were performed using Pearson’s chi-square test for categorical variables and the Wilcoxon rank-sum test for continuous variables. Subgroup analyses were conducted across strata of hospital THA volume, surgical indication, and implant fixation as defined above.

We performed univariate and multivariable logistic regression analyses to identify factors associated with ≥ XL head use. The multivariable model included surgical indication, fixation method, hospital surgical volume, sex, age, body mass index, and Elixhauser comorbidity score. Results are presented as odds ratios (OR) with 95% confidence intervals (CI).

Cumulative revision rates were estimated using the Kaplan–Meier method with 95% log-log confidence intervals and stratified by head length, hospital volume, indication and fixation. Differences between survival curves were assessed using the log-rank test. All statistical analyses were performed using R version 4.4.2 (R Foundation for Statistical Computing), and a p-value < 0.05 was considered statistically significant.

## Results

When stratified by hospital volume, the use of ≥ XL femoral heads demonstrated a statistically significant decreasing trend with increasing institutional volume. In low-volume hospitals, 5.4% of implants were ≥ XL, compared with 4.5% in medium-volume centers and 3.0% in high-volume hospitals (*p* < 0.001). The detailed distribution of ≥ XL versus XS–S–M–L heads across hospital volumes is presented in Table 5.

**Table 5:** Distribution of femoral head lengths by hospital surgical volume

Variable	0–250	251–500	≥ 501	*p*-value^2^
≥ XL, *N* = 26,186^1^*	15,516 (5.4%)	5,448 (4.5%)	4,139 (3.0%)	< 0.001
XS-S-M–L, *N* = 535,815^1^*	272,331 (94.6%)	115,345 (95.5%)	134,026 (97.0%)	

*Hospital volume data were unavailable for 1,083 patients in the ≥ XL group and 14,113 patients in the XS–S–M–L group; these cases were excluded from this analysis.

^1^*n* (%); Median (Q1, Q3)

^2^Pearson’s Chi-squared test; Wilcoxon rank sum test

The use of ≥ XL femoral heads varied significantly by both surgical indication and hospital volume. Usage was consistently higher in lower-volume hospitals (*p* < 0.001), and ≥ XL heads were more frequently used in patients with FNF compared with those with primary OA (8.1% vs. 4.7% in low-volume hospitals). This difference persisted in high-volume hospitals, where ≥ XL head use remained higher for FNF than for OA (5.0% vs. 2.7%, respectively). The detailed distribution of head lengths across hospital volume categories within each indication subgroup is presented in Table 6.

**Table 6:** Use of ≥ XL femoral heads by surgical indication and hospital volume

Indication	Hospital volume	XL Heads (*N*, %)	Total (*N*)	*p*-value
*Femoral Neck Fracture*	≤ 250	2,076 (8.1%)	25,483	< 0.001
	251–500	202 (6.2%)	3,260	
	≥ 501	75 (5.0%)	1,512	
*Primary hip osteoarthritis*	≤ 250	9,762 (4.7%)	207,296	< 0.001
	251–500	4,142 (4.5%)	91,494	
	≥ 501	2,894 (2.7%)	107,794	

In terms of implant fixation, implantation of ≥ XL femoral heads was significantly higher for cementless stems compared with cemented stems in medium-volume (4.8% vs. 3.1%) and high-volume hospitals (3.1% vs. 2.3%, *p* < 0.001), whereas rates were similar between fixation types in low-volume hospitals (5.2% vs. 5.1%). (Table 7)

**Table 7:** Variation in ≥ XL femoral head use across fixation methods and hospital volumes

Implant fixation	Hospital volume	XL Heads (*N*, %)	Total (*N*)	*p*-value
*Cemented*	≤ 250	3,277 (5.1%)	62,881	< 0.001
	251–500	703 (3.1%)	22,501	
	≥ 501	636 (2.3%)	27,500	
*Cementless*	≤ 250	11,324 (5.2%)	217,293	< 0.001
	251–500	4,528 (4.8%)	94,602	
	≥ 501	3,430 (3.1%)	109,708	

In multivariate analysis, several factors were independently associated with increased odds of using a femoral head size ≥ XL. Male sex (OR = 2.13, 95% CI 2.06–2.19, *p* < 0.001), higher BMI categories, particularly BMI ≥ 40 (OR = 1.94, 95% CI 1.77–2.12, *p* < 0.001), higher Elixhauser comorbidity scores (≥ 4; OR = 1.09, 95% CI 1.04–1.15, *p* < 0.001), and surgery performed for FNF (OR = 1.92, 95% CI 1.83–2.02, *p* < 0.001) were all associated with greater likelihood of using ≥ XL heads.

Although unadjusted analyses showed variation in ≥ XL head use according to fixation type and hospital volume, multivariable adjustment demonstrated that cemented fixation was independently associated with slightly higher odds compared with cementless fixation (OR = 1.14, 95% CI 1.09–1.18, *p* < 0.001). Importantly, hospital volume showed a strong inverse association, with procedures performed at centers conducting ≥ 501 THAs annually exhibiting lower odds of ≥ XL head use compared with low-volume hospitals (OR = 0.56, 95% CI 0.54–0.58, *p* < 0.001) (Table 8).

**Table 8:** Predictors of ≥ XL femoral head use in primary THA: univariable and multivariable logistic regression

Characteristic	Univariate			Multivariante		
	OR^1^	95% CI^1^	*p*-value	OR^1^	95% CI^1^	*p*-value
*Sex*						
Female	–	–		–	–	
Male	2.07	2.02, 2.12	< 0.001	2.13	2.06, 2.19	< 0.001
*Age*	0.99	0.99, 0.99	< 0.001	0.99	0.99, 1.00	< 0.001
*BMI*						
Normal (18.5–24.99)	–	–		–	–	
Underweight (< 18.5)	1.05	0.90, 1.21	0.5	0.94	0.77, 1.13	0.5
Pre-obese (25–29.99)	1.15	1.11, 1.20	< 0.001	1.11	1.06, 1.16	< 0.001
Obese 1 (30.0–34.99]	1.29	1.24, 1.34	< 0.001	1.27	1.21, 1.34	< 0.001
Obese 2 [35.0–39.99]	1.54	1.46, 1.63	< 0.001	1.57	1.46, 1.67	< 0.001
Obese 3 [ ≥ 40)	1.91	1.77, 2.05	< 0.001	1.94	1.77, 2.12	< 0.001
*Elixhauser score*						
( < 0)	–	–		–	–	
(0–3)	0.89	0.86, 0.92	< 0.001	0.99	0.95, 1.04	0.8
(≥ 4)	1.11	1.06, 1.15	< 0.001	1.09	1.04, 1.15	< 0.001
*Surgical indication*						
Primary hip osteoarthritis	–	–		–	–	
Fracture	1.94	1.85, 2.02	< 0.001	1.92	1.83, 2.02	< 0.001
*Implant fixation*						
cementless	–	–		–	–	
Cemented	1.02	0.99, 1.05	0.13	1.14	1.09, 1.18	< 0.001
*Annual Hospital THA volume*						
≤ 250	–	–		–	–	
251–500	0.83	0.80, 0.86	< 0.001	0.95	0.91, 0.98	0.004
≥ 501	0.54	0.52, 0.56	< 0.001	0.56	0.54, 0.58	< 0.001

*BMI* Body mass index

**Fig. 1:**
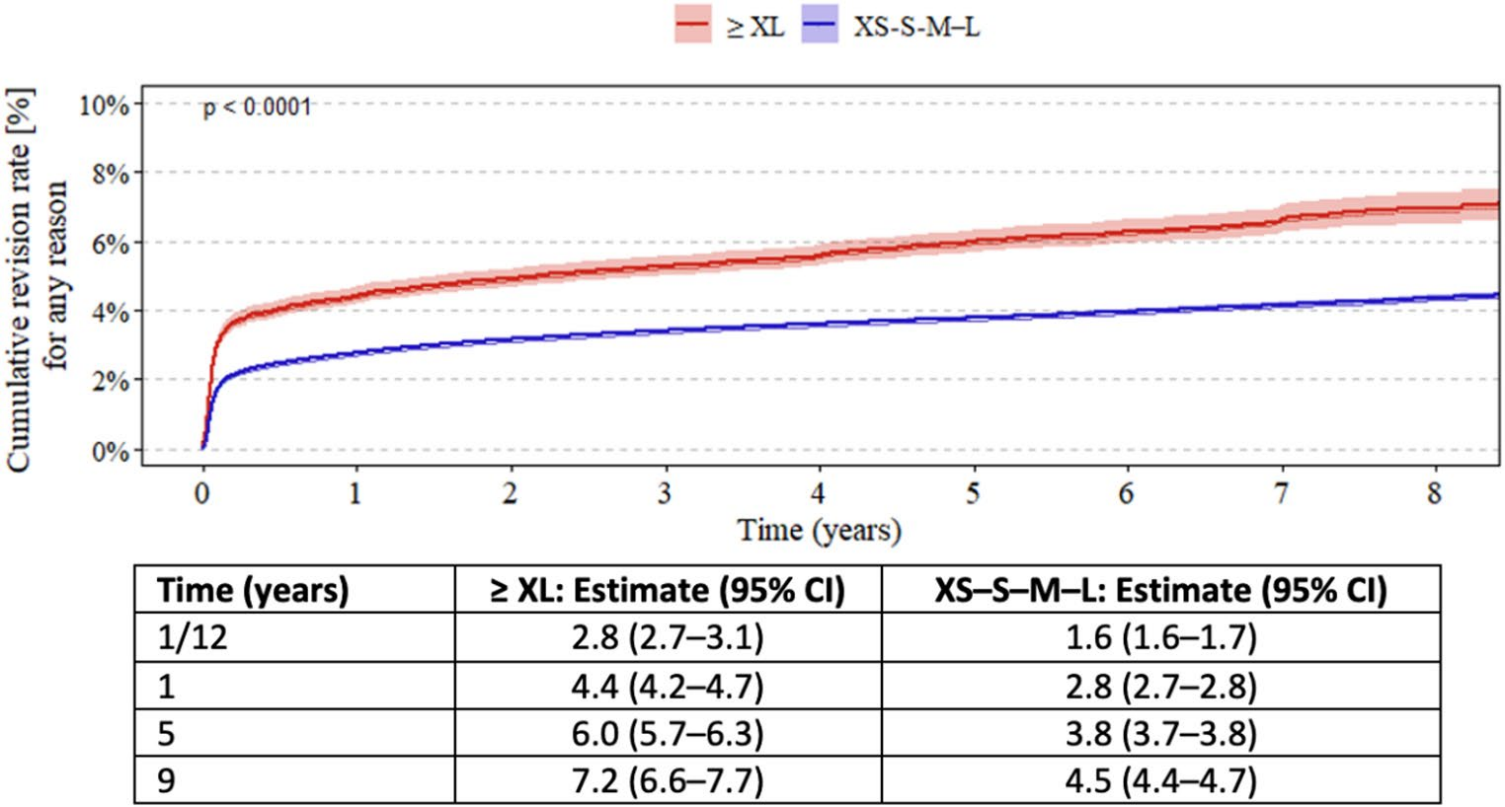
Kaplan–Meier estimates of cumulative revision rates for primary total hip arthroplasty, comparing patients receiving ≥ XL femoral heads with those receiving XS–S–M–L heads. Numerical values are presented as estimates with 95% confidence intervals (CI) at 1 month, and at 1, 5, and 9 years

Kaplan–Meier estimates demonstrated significantly higher revision rates for patients receiving ≥ XL femoral heads compared with those receiving XS–S–M–L heads across all primary THAs. At 1 year, the cumulative incidence of revision was 4.4% in the ≥ XL group compared to 2.8% in the XS–S–M–L group. At 9 years, the cumulative incidence reached 7.2% in the ≥ XL group versus 4.5% in the XS–S–M–L group (Fig. 1).

**Fig. 2:**
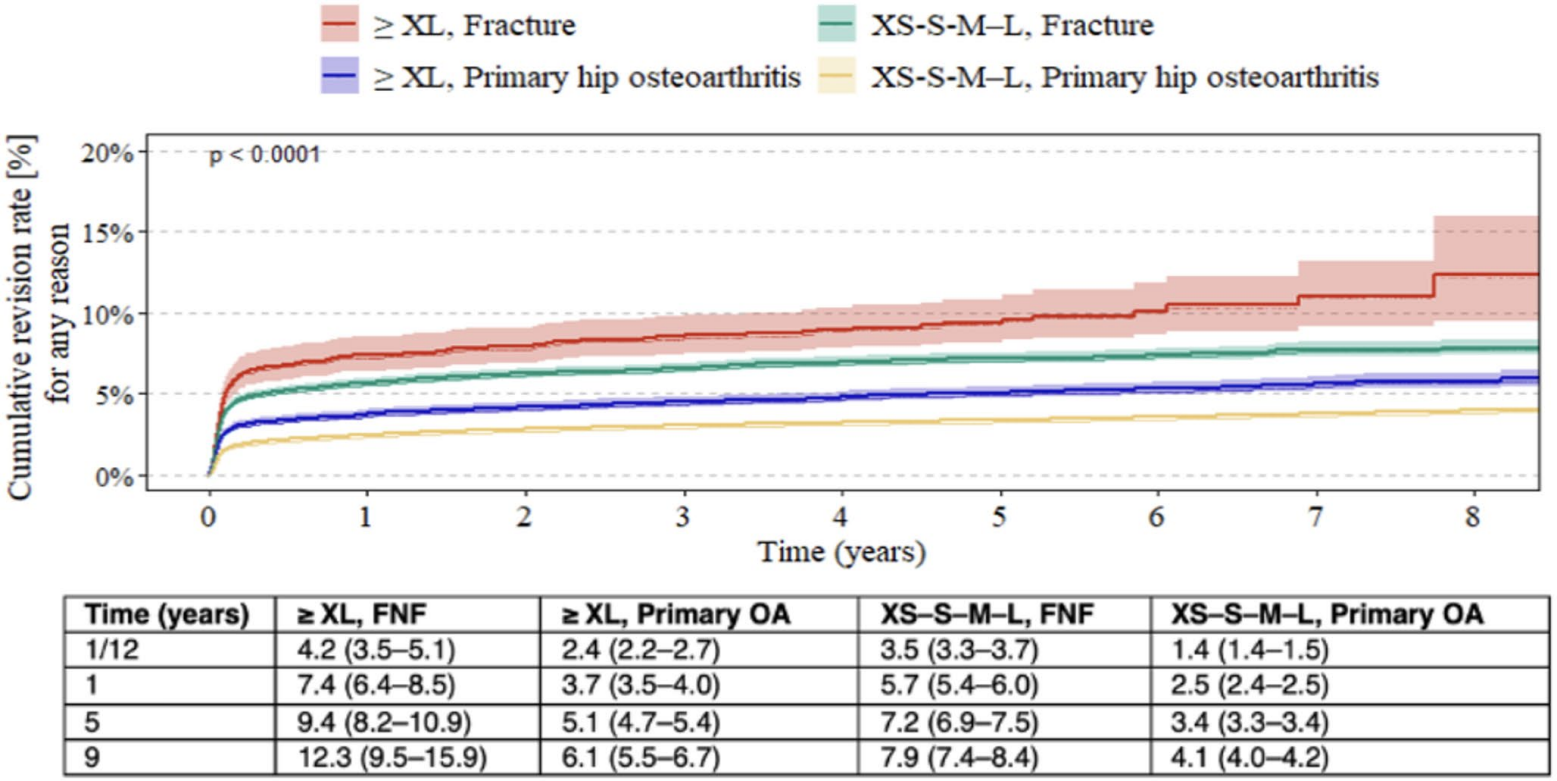
Kaplan–Meier estimates of cumulative revision rates in patients undergoing primary total hip arthroplasty, comparing those with femoral neck fractures and those with primary osteoarthritis, stratified by head length (≥ XL vs. XS–S–M–L). Estimates are shown with 95% confidence intervals. *FNF* femoral neck fracture, *OA* osteoarthritis

Kaplan–Meier estimates showed that within both FNF and primary OA groups, patients receiving ≥ XL heads had higher cumulative revision rates compared to those with XS–S–M–L heads. However, the revision rate in the ≥ XL primary OA group remained lower than that observed in the XS–S–M–L fracture group (Fig. 2).

**Fig. 3:**
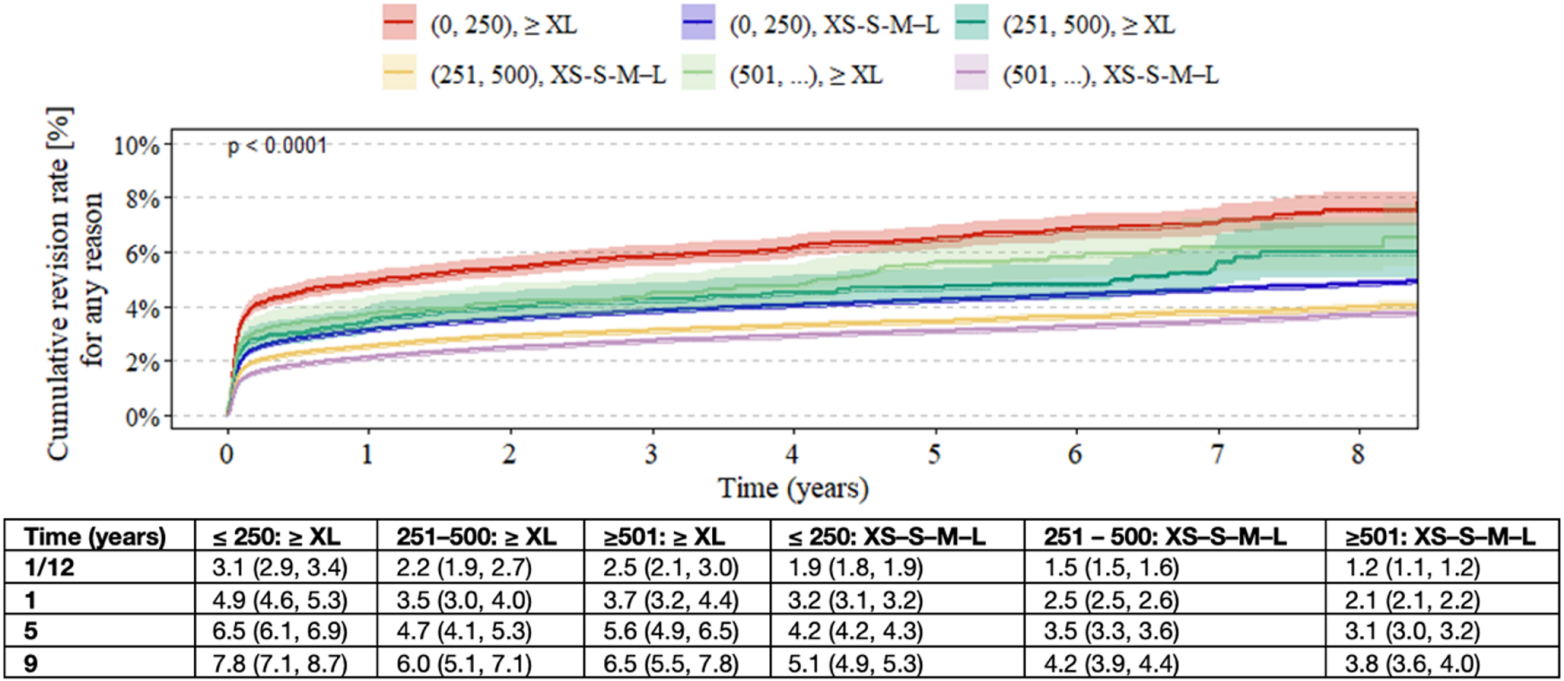
Kaplan–Meier estimates of cumulative revision rates in patients undergoing primary total hip arthroplasty, stratified by annual hospital volume category (≤ 250, 251–500, and ≥ 501 THA per year) and femoral head length (≥ XL vs. XS–S–M–L). Estimates are shown with 95% confidence intervals

In the XS–S–M–L group, the cumulative revision rate increased as hospital volume decreased, rising from 2.1% to 3.2% at 1 year and from 3.8% to 5.1% at 9 years for the highest- versus lowest-volume hospitals, respectively. For ≥ XL heads, low-volume hospitals also had higher cumulative revision rates than high-volume centers (4.9% vs. 3.7% at 1 year and 7.8% vs. 6.5% at 9 years), while hospitals performing 251–500 procedures had the lowest rates overall (3.5% at 1 year and 6.0% at 9 years) (Fig. 3).

**Fig. 4:**
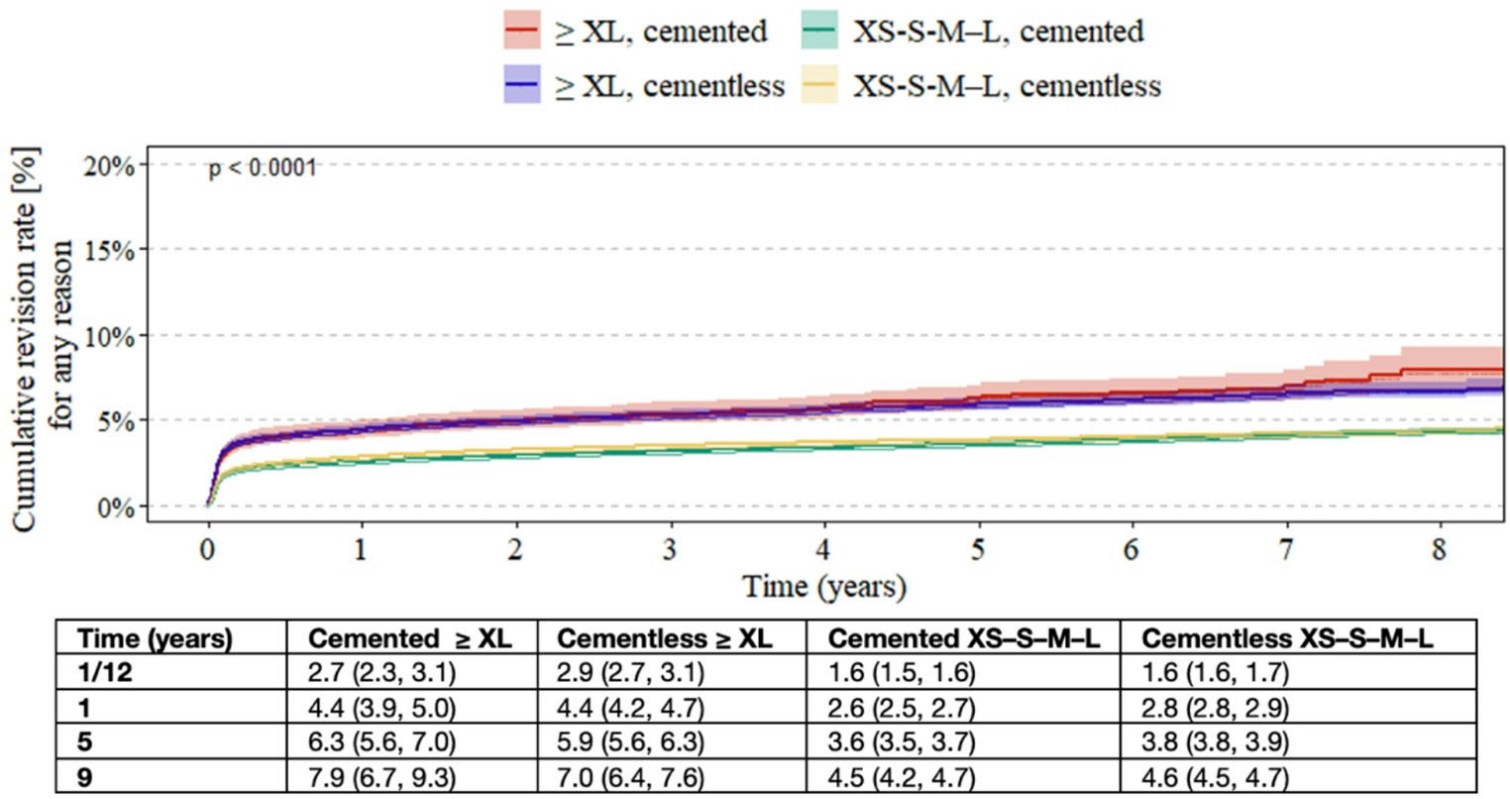
Kaplan–Meier estimates of cumulative revision rates in patients undergoing primary total hip arthroplasty, stratified by femoral head length (≥ XL vs. XS–S–M–L) and fixation method (cemented vs. cementless). Estimates are shown with 95% confidence intervals

Across the follow-up period, ≥ XL heads demonstrated higher cumulative revision rates than XS–S–M–L heads, regardless of fixation method. For ≥ XL heads, rates increased from 4.4% at 1 year to 7.9% at 9 years in the cemented group and from 4.4% to 7.0% in the cementless group, with only small differences between fixation methods. In contrast, the XS–S–M–L group showed lower overall revision rates, ranging from 2.6% to 4.5% for cemented fixation and from 2.8% to 4.6% for cementless fixation over the same period (Fig. 4).

The distribution of revision causes was similar in both groups: infection (≥ XL 23.3% vs. XS–S–M–L 22.2%), missing data (32.5% vs. 31.1%), dislocation (10.5% vs. 10.6%), periprosthetic fracture (10.5% vs. 11.6%), and stem loosening (6.0% vs. 6.2%).

## Discussion

The primary finding of this study is that the use of femoral heads ≥ XL was independently associated with lower hospital primary THA volume, FNF, and, to a lesser extent, with cemented fixation. Furthermore, implantation of ≥ XL heads was associated with higher cumulative revision rates, predominantly driven by early revision.

Prior studies have demonstrated that high surgical volume is linked to a lower risk of complications and improved outcomes [12, 20], likely reflecting differences in infrastructure, experience, and standardization of care processes. Our findings align with this evidence, showing that ≥ XL heads are more frequently used in low-volume compared with high-volume hospitals likely reflecting a greater need for last-resort intraoperative adjustments to optimize factors such as stability. To limit potential bias from patient selection and complex cases, we performed a subgroup analysis restricted to primary OA. This confirmed that the association between hospital volume and ≥ XL head use persisted, with rates of 4.7% in low-volume hospitals compared to 2.7% in high-volume hospitals.

Multivariable logistic regression revealed that FNF was independently associated with the use of ≥ XL heads. The higher use of extra-long heads in FNF cases likely reflects the urgent nature of these procedures, where comprehensive preoperative planning is limited and more extensive soft-tissue dissection may be required [21], potentially necessitating additional intraoperative adjustments such as ≥ XL heads to optimize joint stability.

These results may be partly explained by the trauma setting, where arthroplasties for FNF are performed by either general orthopedic surgeons or fellowship-trained arthroplasty specialists. Given that fellowship-trained surgeons have been shown to achieve more favorable discharge outcomes [22] and lower complication rates, especially with respect to dislocation [23], the higher reliance on ‘last-resort’ intraoperative solutions, such as extra-long heads, may reflect the practice of non-specialists aiming to optimize soft-tissue stability once the prosthesis has been implanted.

Lastly, FNF patients often present with pre-existing muscle weakness or atrophy, which compromises soft-tissue tension and increases their risk of postoperative dislocation, potentially prompting surgeons to favor the intraoperative use of longer heads to enhance joint stability [21, 24].

Although descriptive data analysis suggested that cementless stems were more frequently implanted with XL heads in medium- and high-volume hospitals, multivariate analysis demonstrated that cemented fixation was independently associated with increased odds of ≥ XL head use, albeit of modest magnitude (OR 1.14). This apparent discrepancy likely reflects case-mix differences, as cemented stems are more often selected for older, comorbid, or fracture patients.

After adjusting for confounders, the higher proportion of ≥ XL heads in cemented implants may be related to differences in the final implantation process. While differences between the final position of uncemented femoral stems and the last trial rasps have been investigated in few studies, to the best of the authors’ knowledge, no comparable studies exist for cemented arthroplasty [19, 25, 26]. Hofstaedter et al. [19] reported an average stem protrusion of 2.6 mm (range − 1.5–7.5 mm), with uncemented stems generally sitting more proud than the rasp due to the stem’s coating. This finding is in line with our results and may partly explain the lower need for extra-long heads observed in cementless implants. Moreover these findings may reflect stem design, as some cemented systems lack high-offset options, leading surgeons to use longer heads to restore offset and leg length.

Furthermore the same risk factors linked to revision, including male sex [27], higher BMI [28, 29], and greater comorbidity burden [30, 31] as measured by the Elixhauser score, were also associated with the use of ≥ XL femoral heads, suggesting that XL head implantation may serve as a surrogate marker of higher procedural complexity and revision risk rather than a direct cause.

Our survival analysis demonstrated that ≥ XL femoral heads were associated with significantly higher cumulative revision rates compared with XS–S–M–L heads, with a 57% relative increase at 1 year (4.4% vs. 2.8%), and a persistent gap at 9 years (7.2% vs. 4.5%). This trend was consistent across subgroups. Notably, patients with FNF receiving ≥ XL heads experienced the highest revision rates, reaching 12.3% at 9 years. This could be explained by FNF as an indication per se, which is a well-established risk factor for revision, as demonstrated by Zhao et al., who reported significantly higher 10-year cumulative incidences of all-cause revision (7.1% vs. 4.9%), in THA performed for FNF compared with OA [21].

With respect to the gradual late increase in revisions observed in the fracture subgroup, we emphasize that the following considerations represent our hypothesis. FNF patients are typically older and more fragile and may present with greater baseline instability and compromised soft tissues at the time of implantation. Over time, progressive functional decline, persistent bone fragility, and increased susceptibility to infection in this comorbid population may contribute to cumulative late failures. The continued divergence at longer follow-up therefore likely reflects the intrinsic risk trajectory of this vulnerable subgroup rather than a specific long-term disadvantage attributable to the ≥ XL head.

Furthermore, in accordance with existing literature [11], the revision risk was also influenced by hospital volume, with low-volume centers showing higher cumulative revision rates for both head size categories compared to high-volume centers (7.8% vs. 6.5% at 9 years for ≥ XL heads). However, the use of ≥ XL heads remained a stronger predictor of revision than hospital volume alone, as revision rates for ≥ XL heads exceeded those for XS–S–M–L heads across all hospital volume categories.

Lastly, this study revealed that cemented ≥ XL heads were more likely to be revised compared with cementless ≥ XL heads in the late follow-up period (7.9% vs. 7.0% at nine years).

This association may partly reflect selection bias and unmeasured confounders, including intraoperative decisions to cement in higher-risk, fragile, osteoporotic patients. Supporting this interpretation, in a 1:1 propensity-matched analysis Moore et al. reported higher rates of infection, aseptic loosening, and aseptic revision in patients > 65 years treated with cemented THA compared with cementless fixation [32]. However, in contrast, Rocha et al. found no significant difference in implant survival among patients > 70 years when comparing cemented and cementless fixation [33].

Interestingly, the distribution of revision causes, particularly infection and dislocation, was similar between ≥ XL and XS–S–M–L head groups.

Furthermore, closer inspection of the Kaplan–Meier curves indicates that the increased revision rate associated with ≥ XL heads is predominantly driven by early postoperative failures, followed by only a limited relative increase over time. No specific cause of revision was disproportionately associated with ≥ XL heads, suggesting that the excess risk more likely reflects technical complexity at the time of index surgery, for which ≥ XL head use may serve as a surrogate marker, rather than an inherent implant-related disadvantage. In situations where achieving adequate stability or restoring offset proves challenging, surgeons may resort to longer head lengths as a compensatory strategy. The increased early revision rate may therefore reflect the complexity of the index procedure and associated operative demands rather than a progressive mechanical effect of head length itself.

This study has several limitations inherent to its retrospective, registry-based design. First, the analysis is dependent on the accuracy and completeness of registry data and is therefore susceptible to misclassification, for example in ICD-10 coding, as well as potential underreporting. As participation in the EPRD is voluntary, contributing centers may represent those with greater rigor in data entry. Second, the registry does not provide information on preoperative planning, surgeon experience, intraoperative stability testing, or the rationale for selecting ≥ XL heads, which limits the ability to establish causal relationships. Subgroup analyses were performed to partially mitigate this limitation. Third, although subgroup and multivariable analyses adjusted for sex, age, BMI, Elixhauser score, hospital volume, fixation type, and surgical indication, residual confounding remains possible. Nonetheless, the EPRD captures a broad range of comorbidities, which helps reduce bias. Moreover, we did not perform a separate analysis stratified by femoral head material (ceramic vs. metal), which may influence implant survival. However, as this was not conducted for either ≥ XL or XS–S–M–L head groups, any potential effect is expected to be evenly distributed and unlikely to bias the comparative results. Fourth, similar to findings in total knee arthroplasty where thicker polyethylene inserts were associated with higher revision rates but were interpreted as surrogate markers of intraoperative complexity rather than causative factors, we acknowledge that different manufacturers define “XL” femoral heads differently (length, collar design), which may introduce heterogeneity [34]. However, the objective of our study is not to imply that “XL” heads intrinsically increase revision risk, but rather to suggest that their use may serve as a marker of case complexity and provide an additional clinical indicator when evaluating patients postoperatively. Lastly, hospital volume was defined based on previously published analyses of the EPRD [18]. However, the threshold of 250 procedures per year reflects institutional volume rather than individual surgeon volume, which may introduce bias. Furthermore, 250 procedures per year already represents a relatively high procedural volume and may therefore distinguish high- from very-high-volume institutions rather than low- from medium-volume centers.

## Conclusion

The use of femoral heads ≥ XL was independently associated with lower hospital THA volume, FNF, cemented fixation, male sex, higher BMI, and greater comorbidity burden. Furthermore, implantation of ≥ XL heads was associated with increased revision rates. Rather than representing a causal factor, ≥XL head use appears to serve as a surrogate marker for elevated revision risk.

## Data Availability

No datasets were generated or analysed during the current study.

## References

[1] Sloan M, Premkumar A, Sheth NP (2018) Projected volume of primary total joint arthroplasty in the U.S., 2014 to 2030. J Bone Joint Surg 100:1455–1460. 10.2106/JBJS.17.01617 30180053 10.2106/JBJS.17.01617

[2] Beckers G, Morcos MW, Lavigne M et al (2024) Excellent results of large-diameter ceramic-on-ceramic bearings in total hip arthroplasty at minimum ten-year follow-up. J Arthroplasty 39:3028–3035. 10.1016/j.arth.2024.06.045 38909852 10.1016/j.arth.2024.06.045

[3] Ng KCG, Jeffers JRT, Beaulé PE (2019) Hip joint capsular anatomy, mechanics, and surgical management. J Bone Joint Surg 101:2141–2151. 10.2106/JBJS.19.00346 31800428 10.2106/JBJS.19.00346 PMC7406151

[4] Pitz-Gonçalves LI, Deckard ER, Meneghini RM (2023) Large femoral heads and select dual-mobility bearings are associated with reduced instability in contemporary posterior approach total hip arthroplasty. J Arthroplasty 38:S124–S130. 10.1016/j.arth.2023.02.011 36791889 10.1016/j.arth.2023.02.011

[5] Howie DW, Holubowycz OT, Middleton R (2012) Large femoral heads decrease the incidence of dislocation after total hip arthroplasty. J Bone Joint Surg 94:1095–1102. 10.2106/JBJS.K.00570 22717828 10.2106/JBJS.K.00570

[6] Delbalso CTMG, Tan SC, Lanting BA, Howard JL (2015) Taperosis. Bone Joint J 97–B:911–916. 10.1302/0301-620X.97B7.35149 10.1302/0301-620X.97B7.35149 26130345

[7] Kao Y-YJ, Koch CN, Wright TM, Padgett DE (2016) Flexural rigidity, taper angle, and contact length affect fretting of the femoral stem trunnion in total hip arthroplasty. J Arthroplasty 31:254–258. 10.1016/j.arth.2016.02.079 27094241 10.1016/j.arth.2016.02.079

[8] Kurtz SM, Kocagöz SB, Hanzlik JA et al (2013) Do ceramic femoral heads reduce taper fretting corrosion in hip arthroplasty? a retrieval study. Clin Orthop Relat Res 471:3270–3282. 10.1007/s11999-013-3096-2 23761174 10.1007/s11999-013-3096-2 PMC3773155

[9] Mistry JB, Chughtai M, Elmallah RK et al (2016) Trunnionosis in total hip arthroplasty: a review. J Orthop Traumatol 17:1–6. 10.1007/s10195-016-0391-1 26868420 10.1007/s10195-016-0391-1 PMC4805640

[10] Salmons HI, Karczewski D, Ledford CK et al (2023) Femoral head length impact on outcomes following total hip arthroplasty in 36 millimeter cobalt chrome-on-highly crosslinked polyethylene articulations. J Arthroplasty 38:1787–1792. 10.1016/j.arth.2023.02.031 36805114 10.1016/j.arth.2023.02.031

[11] Glassou EN, Hansen TB, Mäkelä K et al (2016) Association between hospital procedure volume and risk of revision after total hip arthroplasty: a population-based study within the Nordic Arthroplasty Register Association database. Osteoarthr Cartil 24:419–426. 10.1016/j.joca.2015.09.014 10.1016/j.joca.2015.09.014 26432511

[12] Laucis NC, Chowdhury M, Dasgupta A, Bhattacharyya T (2016) Trend toward high-volume hospitals and the influence on complications in knee and hip arthroplasty. J Bone Joint Surg 98:707–712. 10.2106/JBJS.15.00399 27147682 10.2106/JBJS.15.00399 PMC4850659

[13] Siddiqi A, Alamanda VK, Barrington JW et al (2022) Effects of Hospital and surgeon volume on patient outcomes after total joint arthroplasty: reported from the american joint replacement registry. J Am Acad Orthop Surg 30:e811–e821. 10.5435/JAAOS-D-21-00946 35191864 10.5435/JAAOS-D-21-00946

[14] Forssten MP, Mohammad Ismail A, Borg T et al (2022) The consequences of out-of-hours hip fracture surgery: insights from a retrospective nationwide study. Eur J Trauma Emerg Surg 48:709–719. 10.1007/s00068-021-01804-y 34622327 10.1007/s00068-021-01804-y PMC9001198

[15] Ricci WM, Gallagher B, Brandt A et al (2009) Is After-hours orthopaedic surgery associated with adverse outcomes? J Bone Joint Surgery-American Volume 91:2067–2072. 10.2106/JBJS.H.00661 10.2106/JBJS.H.00661 19723981

[16] Jansson V, Grimberg A, Melsheimer O et al (2019) Orthopaedic registries: the German experience. EFORT Open Rev 4:401–408. 10.1302/2058-5241.4.180064 31210976 10.1302/2058-5241.4.180064 PMC6549118

[17] Ravi B, Jenkinson R, Austin PC et al (2014) Relation between surgeon volume and risk of complications after total hip arthroplasty: propensity score matched cohort study. BMJ 348:g3284–g3284. 10.1136/bmj.g3284 24859902 10.1136/bmj.g3284 PMC4032026

[18] Steinbrück A, Grimberg A, Melsheimer O, Jansson V (2020) Einfluss der institutionellen Erfahrung auf die Ergebnisse in Hüft- und Knietotalendoprothetik. Orthopade 49:808–814. 10.1007/s00132-020-03963-z 32885289 10.1007/s00132-020-03963-z

[19] Wier J, Palmer R, Telang S et al (2025) Low-volume surgeons operating at high-volume hospitals have low rates of periprosthetic joint infection after hip and knee arthroplasty. J Arthroplasty 40:1317–1325e4. 10.1016/j.arth.2024.10.136 39515401 10.1016/j.arth.2024.10.136

[20] Zhao AY, Parel PM, Agarwal AR et al (2025) Increased risk of 10-Year revision following total hip arthroplasty for femoral neck fracture. J Arthroplasty 40:688–692. 10.1016/j.arth.2024.09.012 39284393 10.1016/j.arth.2024.09.012

[21] Cao A, Ghanem ES, Cichos KH et al (2023) Comparison between orthopaedic trauma versus arthroplasty fellowship training on outcomes of total hip arthroplasty for femoral neck fracture. J Arthroplasty 38:S72–S77. 10.1016/j.arth.2023.04.009 37068569 10.1016/j.arth.2023.04.009

[22] Mabry SE, Cichos KH, McMurtrie JT et al (2019) Does Surgeon fellowship training influence outcomes in hemiarthroplasty for femoral neck fracture? J Arthroplasty 34:1980–1986. 10.1016/j.arth.2019.04.038 31104837 10.1016/j.arth.2019.04.038

[23] Woolson ST, Rahimtoola ZO (1999) Risk factors for dislocation during the first 3 months after primary total hip replacement. J Arthroplasty 14:662–668. 10.1016/S0883-5403(99)90219-X 10512436 10.1016/s0883-5403(99)90219-x

[24] Barink M, Meijers H, Spruit M et al (2004) How close does an uncemented hip stem match the final rasp position? Acta Orthop Belg 70:534–539 15669452

[25] Hofstaedter T, Najfeld M, Fessel G et al (2020) Discrepancy of trial rasp and femoral stem relative position within the femoral canal of a coated tapered system: an intraoperative, intrapatient Controlled Study. Arthroplast Today 6:819–824. 10.1016/j.artd.2020.07.032 33015261 10.1016/j.artd.2020.07.032 PMC7522528

[26] Tippimanchai T, Suksathien Y, Sueajui J (2024) Seating of the femoral stem after washing versus un-washing the femoral canal in cementless short stem hip arthroplasty. J Southeast Asian Orthop. 10.56929/jseaortho-2024-0225

[27] Prokopetz JJ, Losina E, Bliss RL et al (2012) Risk factors for revision of primary total hip arthroplasty: a systematic review. BMC Musculoskelet Disord 13:251. 10.1186/1471-2474-13-251 23241396 10.1186/1471-2474-13-251 PMC3541060

[28] Jeschke E, Citak M, Günster C et al (2018) Obesity increases the risk of postoperative complications and revision rates following primary total hip arthroplasty: an analysis of 131,576 total hip arthroplasty cases. J Arthroplasty 33:2287–2292e1. 10.1016/j.arth.2018.02.036 29551304 10.1016/j.arth.2018.02.036

[29] Wagner ER, Kamath AF, Fruth KM et al (2016) Effect of body mass index on complications and reoperations after total hip arthroplasty. J Bone Joint Surg 98:169–179. 10.2106/JBJS.O.00430 26842406 10.2106/JBJS.O.00430

[30] Arias-de la Torre J, Smith K, Dregan A et al (2020) Impact of comorbidity on the short- and medium-term risk of revision in total hip and knee arthroplasty. BMC Musculoskelet Disord 21:447. 10.1186/s12891-020-03455-3 32646395 10.1186/s12891-020-03455-3 PMC7346613

[31] Leopold VJ, Krull P, Hardt S et al (2023) Is elective total hip arthroplasty safe in nonagenarians? J Bone Joint Surg 105:1583–1593. 10.2106/JBJS.23.00092 37624906 10.2106/JBJS.23.00092

[32] Moore MC, Dubin JA, Monárrez R et al (2024) Cemented versus cementless femoral fixation for total hip arthroplasty following osteoarthritis. J Arthroplasty 39:1545–1549. 10.1016/j.arth.2023.12.024 38128624 10.1016/j.arth.2023.12.024

[33] Rocha AC, Somerville LE, Moody PW et al (2025) Cementless versus cemented stems in patients aged 70 years or older undergoing total hip arthroplasty. J Arthroplasty 40:S250–S254. 10.1016/j.arth.2025.02.008 39971208 10.1016/j.arth.2025.02.008

[34] Rajamäki A, Niemeläinen M, Junnila M et al (2023) Thicker polyethylene inserts (≥ 13 mm) increase the risk for early failure after primary cruciate-retaining total knee arthroplasty (TKA): a single‐centre study of 7643 TKAs. Knee Surg Sports Traumatol Arthrosc 31:1018–1025. 10.1007/s00167-022-07189-8 36205761 10.1007/s00167-022-07189-8 PMC9957842

